# “B in IT” - a community-based model for the management of hepatitis B patients in primary care clinics using a novel web-based clinical tool

**DOI:** 10.1186/s41124-017-0031-2

**Published:** 2018-01-04

**Authors:** Debra A. O’Leary, Eleanor Cropp, David Isaac, Paul V. Desmond, Sally Bell, Tin Nguyen, Darren Wong, Jessica Howell, Jacqui Richmond, Jenny O’Neill, Alexander J. Thompson

**Affiliations:** 1 Gastroenterology Department, St Vincent’s Hospital Melbourne, 35 Victoria Parade, Fitzroy, VIC 3065 Australia; 2 GP Liaison Department, St Vincent’s Hospital Melbourne, 35 Victoria Parade, Fitzroy, VIC 3065 Australia; 3 North Richmond Community Health, 23 Lennox Street, Richmond, VIC 3121 Australia; 4 Victorian Infectious Diseases Service, The Royal Melbourne Hospital, 300 Gratten Street, Parkville, Melbourne, VIC 3000 Australia; 5 La Trobe University, The Australian Research Centre in Sex, Health and Society, 215 Franklin Street, Melbourne, VIC 3000 Australia; 6 Department of General Practice, The University of Melbourne, 200 Berkeley Street, Parkville, Melbourne, VIC 3000 Australia; 7 EpiSoft Pty Ltd, Suite 216, 20 Dale Street, Brookvale, NSW 2100 Australia

**Keywords:** Chronic hepatitis B, Shared care, Hepatologists, Specialists, General practitioners, Primary care, B in IT, Compliance, Liver cancer, HCC screening

## Abstract

**Background:**

The current model of care for the treatment of chronic hepatitis B (CHB) in Australia is through specialist Hepatology or Infectious Diseases clinics, and limited accredited primary care practices. Capacity is limited, and less than 5% of Australians living with CHB currently access therapy. Increasing treatment uptake is an urgent area of clinical need. Nucleos(t)ide analogue therapy is safe and effective treatment for CHB that is suitable for community prescribing. We have evaluated the success of a community-based model for the management of CHB in primary care clinics using a novel web-based clinical tool.

**Methods:**

Using guidelines set out by the Gastroenterological Society of Australia, we developed an interactive online clinical management tool for the shared care of patients with CHB in primary care clinics, with remote oversight from tertiary hospital-based hepatologists and a project officer. We call this model of care the “B in IT” program. Suitable patients were referred from the specialist liver clinic back to primary care for ongoing management. Compliance with recommended appointments, pathology tests and ultrasounds of patients enrolled in “B in IT” was assessed and compared to that of the same patients prior to community discharge, as well as a matched control group of CHB outpatients continuing to attend a specialist clinic.

**Results:**

Thirty patients with CHB were enrolled in the “B in IT” program. Compliance with attending scheduled appointments within 1 month of the suggested date was 87% across all 115 visits scheduled. Compliance with completing recommended pathology within 1 month of the suggested date was 94% and compliance with completing recommended liver ultrasounds for cancer screening within 1 month of the suggested date was 89%. The compliance rates for visit attendance and ultrasound completion were significantly higher than the control patient group (*p* < 0.0001) and the “B in IT” patients prior to community discharge (*p* = 0.002 and *p* = 0.039, respectively).

**Conclusions:**

The “B in IT” program’s novel web-based clinical tool supports primary care physicians to treat and monitor patients with CHB. This program promotes community-based care and increases system capacity for the clinical care of people living with CHB.

## Background

Approximately 1 % of Australians are living with chronic hepatitis B (CHB) [1]. If left untreated, hepatitis B-related cirrhosis, liver failure and hepatocellular carcinoma (HCC) result in mortality of up to 25% for people living with CHB [2–4]. In Australia, the second National Hepatitis B Strategy (2014-2017) set targets for diagnosis and care of people living with CHB, including increasing diagnosis rates of CHB to 80%, and increasing the proportion of patients receiving antiviral treatment to 15% (currently < 5%) [5]. The National Strategy highlights the need for primary care to play a central role in CHB monitoring and calls for the development of models of care to increase the involvement of general practitioners (GPs).

The recommended clinical management for the majority of people living with CHB is now protocol-based [6]. The introduction of safe and effective antiviral therapies for CHB, as well as simple clinical protocols for virological monitoring and HCC screening, makes shared care between specialist liver clinics and primary care physicians attractive, with community-based treatment possible for the majority of people with CHB. As of 1 July 2015 antiviral medications for CHB can be dispensed by community pharmacies in Australia, however there remain a limited number of GPs who are accredited prescribers. Shared care arrangements can help overcome this hurdle by promoting management in the primary care setting with clinical oversight and support provided by the specialist. This model will increase system capacity in both primary and tertiary care settings. Another potential advantage of community-based shared care of CHB is the convenience for patients in seeing their GP for review appointments, rather than needing to visit a hospital outpatient clinic. This may improve adherence of stable patients with six monthly blood tests ± liver ultrasounds for cancer screening recommended by the Gastroenterological Society of Australia (GESA) [6].

Published data describing the implementation of community-based shared care models for the treatment and monitoring of patients living with CHB in Australia are lacking. Here we describe the design and implementation of the “B in IT” program, a community-based model for the management of people living with CHB in primary care clinics using a novel web-based clinical tool overseen remotely by a project officer and specialists in tertiary hospitals.

## Methods

### “B in IT” program

**Fig. 1:**
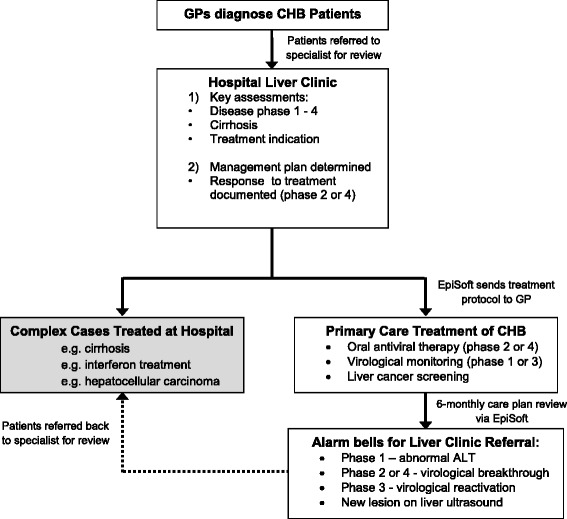
The “B in IT” model of care. Following routine diagnosis at a community GP clinic and referral to the hospital liver clinic for assessment, patients with CHB suitable for discharge back to their GP were identified. For patients who chose to be monitored by their GP, compliance with recommended tests and review appointments was monitored remotely by hospital staff via the EpiSoft web-based clinical guide. Alarm bell messages built into each electronic treatment protocol were used to identify progression of disease and signal if and when a GP should refer the patient back to the hospital liver clinic

The “B in IT” model of care is outlined in Fig. 1. For new patients, following a standard GP referral to a specialist liver clinic, the patient’s CHB phase of disease is established over several consultations and the need for oral antiviral therapy ± HCC screening is determined. As outlined in the GESA algorithm for CHB management [6], there are four recognized phases of disease for CHB. Phases 1 and 3 are monitored by twice yearly serum alanine transaminase (ALT) testing and annual hepatitis B deoxyribonucleic acid (HBV DNA) testing, whereas patients with CHB phases 2 or 4 (characterized by elevated and/or fluctuating ALT and viral load) have these blood tests every 3 – 6 months while receiving antiviral therapy. Patients in any phase of disease who are at high risk of HCC (including Asian males > 40 years of age, Asian females > 50 years of age, those with cirrhosis or a family history of HCC) are also monitored via twice yearly liver ultrasound. Patients with cirrhosis or HCC, patients planned for interferon treatment, patients with a history of antiviral drug resistance, or people with complex medical co-morbidities, are not eligible for the “B in IT” program, and must remain in the care of the specialist liver clinic. All other patients with CHB attending the specialist liver clinic are eligible for the “B in IT” program and can elect to have ongoing care provided by their participating GP. Documented antiviral response is necessary prior to discharge of phase 2 and 4 patients.

EpiSoft cloud-based software was selected for the “B in IT” program because it enables GPs to monitor and treat CHB patients in the community, while still allowing for continued oversight by hepatologists and clinical nurse consultants, as part of a team care arrangement. The “B in IT” treatment protocol outlines when appointments, tests and prescriptions are due, with recall and reminder systems in the event of missed appointments. The system also includes “alarm bell” triggers for specialist review to inform GPs when a patient should be referred back to the specialist liver clinic. For patients being monitored in phase 1 or 3 of disease these triggers are an increase in ALT > 1.5 times the upper limit of normal, an increase in hepatitis B viral load > 2000 IU/ml (phase 3), an increase in fibroscan score > 2.5 kPa (or > 10 kPa score), or a new focal liver lesion detected via ultrasound. For patients being treated with oral nucleos(t)ide analogue therapy in phase 2 or 4 of disease the “alarm bell” triggers are an increase in hepatitis B viral load >10-fold (or from undetectable to detectable), an increase in serum creatinine or decrease in serum phosphate levels (in the case of tenofovir disoproxil fumarate therapy), a decrease in renal function, an increase in fibroscan score > 2.5 kPa (or > 10 kPa score), or a new focal liver lesion detected via ultrasound.

### EpiSoft

**Fig. 2:**
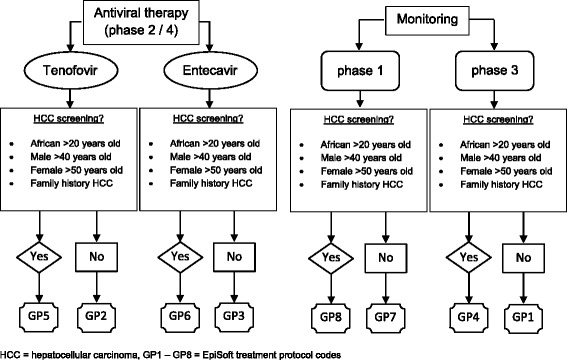
Algorithm for selection of “B in IT” shared care treatment protocols. A hospital liver specialist would assess each patient with CHB to determine their phase of disease, and whether antiviral medication was required. Age, ethnicity and family history of HCC would determine whether twice yearly liver ultrasound was required for HCC screening. This allowed for selection of the most appropriate GP-managed treatment protocol (GP1 – GP8) for those patients who chose to be monitored by their community-based GP. EpiSoft was used to send electronic treatment protocols into the relevant practice management software via secure messaging

Using guideline recommendations for the treatment and monitoring of CHB [6], custom “B in IT” treatment protocols of 12 month duration were developed within the EpiSoft database framework to incorporate monitoring with or without twice yearly abdominal ultrasound for liver cancer screening, and with or without prescription of oral antiviral therapies. Fig. 2 outlines the algorithm for selection of the most appropriate “B in IT” shared care treatment protocol for a patient with CHB. EpiSoft was used to send treatment protocols into the relevant practice management software via secure HealthLink health level 7 (HL7) messaging in portable document format (PDF) that could be saved to the existing patient record at the primary care clinic. A hyperlink within the document allowed GPs to login to EpiSoft (via username and password or Medicare’s National Authentication Service for Health (NASH) Public Key Infrastructure (PKI) token). Incorporation of goals and actions and electronic signatures of care team members enabled GPs to use this documentation to create Medicare billable GP chronic disease management plans and team care arrangements for patients participating in the “B in IT” program. Checkboxes allowed GPs to acknowledge when a patient had attended their appointment and latest ALT and (HBV DNA) test results (viral load) were manually entered within the patient’s treatment protocol page. Checkboxes were used by GPs to indicate if any new focal liver lesions had been identified via ultrasound (where applicable). GPs could print pathology and radiology test request forms as PDF documents directly from EpiSoft, as well as prescriptions for antiviral medication (if required).

“B in IT” treatment protocols allowed secure electronic messaging between all care team members (e.g. when GPs wished to ask the liver specialist a question about a patient’s test results), and email notification was used to alert clinicians of new messages in EpiSoft. Whenever new data was entered, the updated version of the treatment protocol was sent via HL7 messaging from EpiSoft into the GP’s practice management software. Patients could be reminded of upcoming appointments by short message service (SMS) when mobile phone numbers were provided. Reminders were sent three weeks prior to the suggested appointment date, allowing enough time for completion of recommended tests prior to GP review. A variety of user profile types were established within EpiSoft – program manager, hepatologist, clinical nurse consultant, GP and practice nurse. Access privileges varied between user types such that individual GPs and practice nurses could only see data from their own clinic’s patients, whereas the program manager, hepatologists and clinical nurse consultants could access shared care records for all patients who had previously attended their hospital outpatient clinic (discharged to several different GP clinics).

### Primary care site selection

Several GP clinics were identified as referring high numbers of patients with CHB by auditing the St Vincent’s Hospital Melbourne (SVHM) outpatient liver clinic attendance records from June 2012 to July 2013 and cross-referencing against pathology testing for hepatitis B. Practice managers, practice nurses and GPs of four high referring clinics were invited to participate in shared care of their CHB patients. On-site group education sessions regarding diagnosis and management of CHB were conducted by specialists and one-on-one training in the use of the “B in IT” web-based tool was provided by the project officer.

### Specialist engagement

Specialists working within the SVHM outpatient liver clinic with high CHB patient caseloads and who were already caring for patients referred by participating GP clinics were asked to participate in the “B in IT” program. No financial compensation was given to participating specialists, as the time required to review a shared care patient record took an average of less than 5 min every 6 months. This allowed specialists extra time to see CHB patients with more complex/advanced liver disease in a busy public hospital outpatient clinic.

### “B in IT” patient enrolment

CHB patients identified as being referred to the SVHM outpatient liver clinic by participating GP clinics had their records physically flagged prior to their next appointment, and specialists were emailed by the project officer asking them to consider the patients’ eligibility for shared care. Specialists noted in the electronic medical records which CHB patients were suitable and would prefer to see their GP for ongoing treatment/monitoring visits. Since the same level of care was to be continued with their GP (with specialist oversight), verbal consent from participants was adequate for enrolment.

### Control patient identification

A database of all CHB patients attending SVHM outpatient liver clinic was used to identify a control patient cohort, matched in gender, ethnicity, age, treatment type. Two control patients (people continuing to attend the liver clinic) were selected for each “B in IT” patient. These control patients were selected randomly and could be referred from any GP clinic, so may never have been approached regarding participation in the shared care program.

### Role of the project officer

A central project officer is necessary to manage the “B in IT” system. The project officer identified potential shared care patients attending participating GP clinics and SVHM specialist liver clinic by means of auditing appointment attendance from 2012 to 2013 and checking medical records of all new referrals to the liver clinic. The project officer created the custom CHB “B in IT” treatment protocols within EpiSoft, added all new patient records and linked them to the appropriate specialist, GP and treatment protocol based on clinician notes. Triggering of SMS reminders for each patient’s appointments and auditing for compliance was also performed by the project officer using EpiSoft. The project officer reviewed all test results entered by shared care GPs and ensured that specialist and hepatitis nurse consultant care team members co-signed all care plans within EpiSoft following patient review with their GP. Reminder emails were sent from the project officer to GP clinics when “B in IT” patients were overdue for review, prompting their recall. The project officer also acted as a liaison between clinicians and the software provider regarding any software problems that could not be resolved independently.

### Data collection and assessment of compliance

A reporting tool was built into EpiSoft, allowing for real-time monitoring of compliance with recommended dates for review appointments, pathology and ultrasound testing. Data was exported as a .csv file to enable custom sorting by variables such as treating GP, last appointment date or next appointment date, and test results were flagged as overdue if pending more than 6 months (ALT and ultrasound) or 12 months (HBV DNA level). Compliance of “B in IT” patients was assessed at two levels, the proportion of patients who attended a GP review visit and completed recommended tests within 1 month of the date suggested on their treatment protocol, and the proportion of patients who did so within 3 months of the suggested date. Patients were deemed lost to follow up at a GP clinic if they could not be contacted by phone or mail for 3 months after their suggested review date. Patients were deemed to be lost to follow up at a specialist liver clinic if they failed to attend three scheduled appointments. Due dates of recommended appointments and tests were included in compliance assessments when a patient was deemed lost. When comparing the compliance of “B in IT” patients before and during their participation in community-based care, patient data were excluded from the analysis if patients had only attended one specialist appointment. Any visit attended in the specialist liver clinic setting was deemed compliant (even if rescheduled several months later). Cancelled appointments were excluded from the analysis.

### Outcomes

The primary endpoint assessed was compliance of “B in IT” patients with completion of pathology and ultrasound tests every 6 months, and attendance at their community GP clinics for shared care visits to review these results within 1 month of the recommended date. The secondary endpoint assessed was compliance of “B in IT” patients with test and visit completion within 3 months of the recommended date. Additional findings noted were enrollment rate, impact on outpatient liver clinic service and comparison of “B in IT” patient compliance with that of the same patient group prior to their enrolment in shared care. For further comparison of compliance, “B in IT” participants were compared to a control cohort of patients continuing to attend the hospital liver clinic - two control patients were selected for each “B in IT” patient, and groups were matched for gender, age, ethnicity and treatment type.

### Statistical analysis

Parametric data is reported as mean ± standard deviation. Categorical data is reported as number (percentage). Exploratory, bivariate analyses of outcome variables were conducted using parametric or non-parametric tests as appropriate. Differences between characteristics of patient groups were assessed using two-tailed t-tests and two-tailed Fisher exact probability tests. Statistical differences between compliance of patient groups were assessed using the two-tailed Fisher exact probability test. For repeated measures testing in the B in IT group before and after enrolment in shared care, McNemar’s test was used to determine statistical differences in compliance for visit attendance, pathology and ultrasound completion. A two-tailed significance threshold of *p* < 0.05 was used throughout. Analyses were performed using Stata version 12.1 (STATAcorp, Texas US).

## Results

**Fig. 3:**
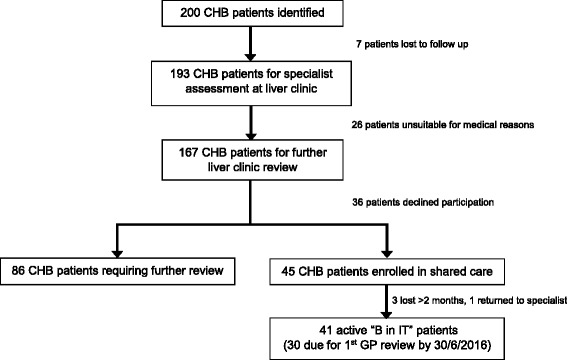
Identification of CHB patients suitable for “B in IT” shared care. All patients with CHB referred to the hospital liver clinic by the four participating “B in IT” GP clinics between August 2013 and June 2016 were assessed for their suitability for shared care. Patients were deemed lost to follow up at the liver clinic if they failed to attend three scheduled appointments. Undetermined phase of disease, complex co-morbidities, history of HCC or cirrhosis were all reasons why patients were deemed unsuitable

Two hundred patients with CHB attending SVHM outpatient liver clinics between August 2013 and June 2016 were referred by GPs from the four clinics participating in the “B in IT” program. As outlined in Fig. 3, 86 of the 167 potentially suitable patients with CHB still required further review at the time of this publication to determine phase of disease, leaving 81 patients with CHB eligible for enrolment in shared care. 45 (56%) patients provided consent to participate in the “B in IT” program. Of the 36 patients with CHB who declined to participate in shared care, only seven specified a reason. Six patients stated that the wait time from check-in until seeing a clinician was shorter at the hospital liver clinic than that at their community GP clinic (all six of these patients attend the same GP clinic), and one patient stated that the hospital liver clinic was conveniently located on the way to their workplace.

Table 1 outlines the clinical features of the 30 patients enrolled in “B in IT” due for their first GP-led monitoring visit by the end of June 2016. Almost two thirds (63.3%) of the patients were female, the average patient age was 55.6 years. The majority of “B in IT” patients were Asian (80%), 40% of patients had previously attended more than ten liver clinic appointments and 80% of patients were in phase 1 or 3 of disease, requiring monitoring only. Eighty three percent of “B in IT” patients also required liver cancer surveillance, involving twice yearly ultrasound as per clinical guidelines [6]. Table 1 also describes the demographics of a group of 60 control CHB patients still attending the SVHM outpatient liver clinic, matched 2:1 to “B in IT” patients for age, gender, ethnicity and treatment type. One of the only statistically significant differences between the patient groups was that 78.3% of control patients had experienced a break in specialist care of more than 1 year compared to only 40% of “B in IT” patients (*p* = 0.0005 Fisher exact test). Also, fewer control patients had been seen in liver clinic for less than 1 year (*p* = 0.005 Fisher exact test).

**Table 1:** Patient Demographics

Characteristic	“B in IT” patients	Control patients	*P* values
Total Number	30	60	
Female	19 (63.3%)	38 (63.3%)	1.000
Male	11 (36.7%)	22 (36.7%)	1.000
Age (years) mean ± SD	55.6 ± 12.6	55.3 ± 11.6	0.985
Female	53.8 ± 10.7	54.0 ± 10.4	0.867
Male	58.6 ± 15.4	57.6 ± 13.4	0.889
Ethnicity			
Asian	24 (80%)	48 (80%)	1.000
Sub-Saharan African	3 (10%)	6 (10%)	1.000
European	2 (6.7%)	4 (6.7%)	1.000
Pacific Islander	1 (3.3%)	2 (3.3%)	1.000
Preferred Language			
English	12 (40%)	30 (50%)	0.502
Vietnamese	6 (20%)	14 (23.3%)	0.794
Mandarin	4 (13.3%)	7 (11.7%)	1.000
Hakka Timorese	3 (10%)	1 (1.7%)	0.106
Cantonese	2 (6.7%)	4 (6.7%)	1.000
Other	3 (10%)	4 (6.7%)	0.682
Liver Clinic Visits			
< 5	10 (33.3%)	11 (18.3%)	0.185
5-10	8 (26.7%)	13 (21.7%)	0.791
> 10	12 (40%)	36 (60%)	0.116
Breaks in care (> 1 year)			
N/A (seen < 1 year)	9 (30%)	4 (6.7%)	0.005^a^
0	12 (40%)	47 (78.3%)	0.0005^a^
1	7 (23.3%)	6 (10%)	0.115
2	1 (3.3%)	3 (5%)	1.000
> 2	1 (3.3%)	0 (0%)	0.333
HBeAg Status			
Negative	29 (96.7%)	57 (95%)	1.000
Positive	1 (3.3%)	3 (5%)	1.000
Phase of disease			
1 – immune tolerance	0 (0%)	0 (0%)	1.000
2 – immune clearance	1 (3.3%)	3 (5%)	1.000
3 – immune control	23 (76.7%)	44 (73.3%)	0.802
4 – immune escape	5 (16.7%)	9 (15%)	1.000
Cleared (HBsAb positive)	1 (3.3%)	4 (6.7%)	0.661
Cirrhosis	0 (0%)	0 (0%)	1.000
Treatment type			
Monitoring	24 (80%) – 20 with HCC screening	48 (80%) – 40 with HCC screening	1.000
Antiviral therapy	6 (20%) – 5 with HCC screening	12 (20%) – 10 with HCC screening	1.000
Entecavir	5 (16.7%)	10 (16.7%)	1.000
Tenofovir	1 (3.3%)	2 (3.3%)	1.000

*SD* standard deviation, *N/A* not applicable, *HBeAg* hepatitis B e antigen, *HBsAb* hepatitis B surface antibody; ^a^statistically significant

Adherence to clinical monitoring and attendance of the 30 patients enrolled in “B in IT” is summarized in Table 2. Eighty three percent of patients attended their first shared care GP visit within 1 month of the suggested date and 87% of all shared care visits scheduled up until the end of 2016 (115 in total) were attended within 1 month of the suggested date. Adherence to the recommended pathology testing schedule within 1 month of the suggested date was 90% at the first shared care GP visit and 94% across all 115 review visits. Compliance of the 24 “B in IT” patients requiring twice yearly liver ultrasounds for cancer screening was 83% at the first visit and 89% across all visits. No significant improvement in adherence was noted if compliance was defined as completion up to 3 months after the suggested date.

**Table 2:** “B in IT” patient compliance

	Attendance 1^st^ visit	Attendance overall	Pathology 1^st^ visit	Pathology overall	Ultrasound 1^st^ visit	Ultrasound overall
Total scheduled	30	115	30	115	24	84
Completed +/− 1 month	25 (83.3%)	100 (87.0%)	27 (90.0%)	108 (93.9%)	20 (83.3%)	75 (89.3%)
Completed +/− 3 months	26 (86.7%)	109 (94.8%)	27 (90.0%)	111 (96.5%)	21 (87.5%)	79 (94.0%)

The percentage of “B in IT” patients compliant with twice yearly visit attendance and completion of pathology tests and ultrasounds (as outlined in their shared care treatment protocols) was compared with that of the same patient group prior to their enrolment in shared care, as well as the control CHB group still attending SVHM liver clinic (see Table 3). Compliance when attending the specialist liver clinic was defined as completion of two liver function tests per year, two liver ultrasounds per year (if necessary), and attendance of all scheduled visits. Compliance of the “B in IT” patients with visit attendance was significantly improved following their enrolment in shared care, with only 50% of patients attending scheduled appointments at liver clinic compared to 86.7% attending twice yearly GP shared care appointments (*p* = 0.002). A higher percentage of “B in IT” patients also completed timely liver ultrasounds – 87.5% overall when enrolled in shared care versus only 26.3% of these patients completing two liver ultrasounds per year prior to community discharge (*p* = 0.039). A significant improvement was also seen in the percentage of “B in IT” patients completing two pathology tests per year - 90.0% overall when enrolled in shared care versus 57.7% when attending the liver clinic (*p* = 0.005).

**Table 3:** Comparison of compliance between patient groups

	Attendance compliance^a^		Pathology compliance^b^		Ultrasound compliance^c^	
	1st year	overall	1st year	overall	1st year	overall
“B in IT” patients	83.3%	86.7%	90.0%	90.0%	87.5%	87.5%
“B in IT” patients prior to discharge	63.3% *P* = 0.083	50.0%^d^ *P* = 0.002	66.7%^d^ *P* = 0.020	57.7%^d^ *P* = 0.005	55.6%^d^ *P* = 0.031	26.3%^d^ *P* = 0.039
Control patients	68.3% *P* = 0.205	40.0%^d^ *P* < 0.0001	80.0% *P* = 0.369	63.3%^d^ *P* = 0.011	47.7%^d^ *P* = 0.002	10.0%^d^ *P* < 0.0001

^**a**^Attendance of all scheduled visits; ^**b**^completion of two liver function tests per year; ^**c**^completion of two liver ultrasounds per year; ^d^statistically significant difference from “B in IT” patient group

A similar pattern of compliance was observed for the control CHB patient group as the “B in IT” patients prior to their enrolment in shared care, with compliance decreasing after the first year of attendance. Only 63.3% of control patients completed twice yearly liver function tests compared to 90% of “B in IT” patients (*p* = 0.011) and only 40% of control CHB patients attended all visits scheduled, compared to 86.7% of “B in IT” patients (*p* < 0.0001). Again, the most dramatic effect of the “B in IT” program was seen when comparing HCC screening twice yearly liver ultrasounds – only 10% of control CHB patients were compliant overall compared to 87.5% of “B in IT” patients (*p* < 0.0001). This difference could also be seen during the first year of liver clinic attendance, with most control CHB patients (70.8%) only having one liver ultrasound per year, even though the GESA recommendation for CHB patients at high risk of HCC is twice yearly liver ultrasound [6].

Qualitative evaluation of participating GPs and specialists was performed using unstructured interview. Feedback from eight participating GPs was positive - GPs found the chronic disease management plan documentation and secure electronic messaging with specialists useful. Two GPs had a “B in IT” patient case load < 2 and reported some difficulty recalling how to use Episoft’s web-based clinical tool, as each patient is only seen twice a year, so often had to contact the project officer and refer to their workflow user guide. The four GPs with “B in IT” patient caseloads > 5 soon became familiar with the web-based tool. All GPs reported feeling more confident in managing CHB since participating in the “B in IT” program, but still liked to have specialist oversight.

Feedback from participating specialists was similar – the more “B in IT” patients seen, the more familiar the clinicians are with use of the web-based tool. Specialists like the clinical summary view of latest pathology and imaging test results, which allows them to quickly review patient status and co-sign chronic disease management plans. Specialists were reliant on the project officer reminding them which patient records are due for review (which patients have recently seen their GP), and GPs were reliant on the project officer reminding them which patients are due for appointments.

At the time of this publication, 45 patients had been enrolled in the “B in IT” program (analysed compliance data is limited to those patients who completed their first shared care visit by end of June 2016), improving access to specialist care of an additional 45 patients with CHB who otherwise may have had their care delayed. This equates to two SVHM outpatient liver clinics per year – encompassing the time of four liver specialists per 3 h clinic. A project officer working 20 h per week has the capacity to oversee shared care of “B in IT” patients from approximately 10 GP clinics, assuming a maximum of 20 patients per GP clinic (up to a total of 200 patients). Expansion of the “B in IT” program greater than this would require an increase in the number of hours worked by one or several project officers.

## Discussion

We have successfully piloted a shared care program for community-based management of people living with CHB using a novel web-based clinical platform – the “B in IT” program. Uptake of “B in IT” shared care to-date has depended upon multiple factors - specialist recommendation to the patient, the existing relationship of the patient with their GP and specialist, convenience in location of the clinic, and languages spoken by the clinicians. For clinicians to find the “B in IT” program familiar and easy to use they need to have a high case load, as each patient is only seen once every 6 months. Compliance of community-based “B in IT” patients has met our expectation and been very good in comparison to patients with CHB attending hospital outpatient liver clinics, since people will generally have less travel time and shorter waiting times per visit when attending their local GP clinic. People also attend community clinics for a variety of other health reasons, so can be reminded of an upcoming CHB review when attending for a different reason. The improvement in compliance with regular ultrasound screening for liver cancer when patients were tracked through the “B in IT” program was drastic. Systematic measures to monitor compliance are required given that liver cancer incidence rates in the state of Victoria are increasing by more than 4% per year in both men and women, and CHB is a significant cause [7]. The “B in IT” program is such a mechanism and is currently being expanded to incorporate patients from additional GP clinics in Melbourne. Early detection of cirrhosis and prevention of liver cancer is difficult to quantify, but every case prevented saves the government hundreds of thousands of dollars in medical costs, with the annual cost of care being > $18,000 for each case of CHB-related HCC and a liver transplant costing > $150,000 [8].

Conservatively at least 25% of CHB patients seen at SVHM could be monitored in the community by their local GP (those in phase 3 of disease) if this program was expanded to include all GPs referring to the SVHM liver clinic. This proportion would be 40% (estimated from an audit of SVHM clinic lists performed during this study) if we include GPs prescribing antiviral medication in the community for treatment of stable phase 2 and 4 patients. There are currently 700 CHB patients seen at the SVHM outpatient liver clinic, which equates to 175-280 potential “B in IT” patients. Based on our observed enrolment rate, even if only 56% of eligible patients consented to participate in the “B in IT” program (98-156 people), this would still significantly increase involvement of GP clinics in CHB treatment and monitoring in Victoria, as called for in the second National Hepatitis B Strategy.

Another benefit of the “B in IT” program is the ability for GPs to send electronic messages to the other care team members, querying unexpected test results, if/when to refer back to liver clinic, or informing the project officer that a patient is overdue due to overseas travel. To-date more than 30 messages have been sent from participating GPs and responses from the care team are obtained within a week. This easy method of communication helps prevent unnecessary referrals back to liver clinic, while allowing specialists to maintain clinical oversight of “B in IT” patients and building capabilities in the primary care workforce for the treatment and monitoring of CHB. Confounders for B in IT attendance were not rigorously assessed in this study, however the trends appear positive for the program having impact.

A limitation of the “B in IT” program to-date is the small number of GP clinics engaged. Larger numbers of patients and clinicians will be needed to more accurately assess the success of this shared care model. As noted in the results, there were also fewer patients seen for less than a year in SVHM liver clinic in the control group compared to the “B in IT” group, so observation of the “B in IT” patients for several years will be necessary to see if their compliance remains high over time. The project officer role is required for this model of care, to ensure that clinician time is focused on the management of patients, not the day-to-day administrative tasks. So if patient and clinician numbers grow for the “B in IT” program the workload of the project officer will grow.

We will continue to monitor the success of the “B in IT” program via compliance of patients with recommended visits, pathology and imaging tests. Satisfaction of GPs and patients will also be determined via a survey. If employed as a long-term model of care, the “B in IT” program could dramatically increase the capacity for treatment of patients with complex cases of CHB at hospital liver clinics. Simplified treatment protocols have also been created within EpiSoft for use within specialist liver clinics to track compliance of all patients with CHB who require twice yearly HCC screening, and this is currently being trialed at SVHM. Monthly audit reports are being generated and any CHB patient overdue for HCC screening (>7 months since last liver ultrasound), and who does not already have a liver clinic appointment booked, is recalled via mailing test request forms with an appointment letter. Over the past 12 months 70% of overdue CHB patients have re-engaged with the liver clinic after first recall. Observation over the coming 6 months is required to determine if additional patients re-engage with the hospital’s liver clinic after second recall.

## Conclusions

The “B in IT” program for shared care of patients with CHB has been established, enabling GPs to treat and monitor patients with CHB in the community, with oversight from a project officer, hepatologists and nurse consultants in tertiary hospitals using EpiSoft. A centralized, secure cloud-based database allows for remote auditing of patient compliance against recommended appointment dates and pathology and ultrasound tests. Compliance of “B in IT” patients has been > 85% to-date, proving this model of care can be successful. With government support and wide-reaching organisations such as primary health networks, the “B in IT” program would be amenable to broad use across the state of Victoria (and throughout Australia) for the treatment of patients with CHB. The “B in IT” program contributes towards the aims of the second National Hepatitis B Strategy to increase treatment uptake to 15% and is also aligned with the government’s objectives to implement eHealth processes in the primary care setting and promote innovation and workforce development within the healthcare sector.

## Abbreviations

ALT: Alanine transaminase
CHB: Chronic hepatitis B
DNA: Deoxyribonucleic acid
GESA: Gastroenterological society of Australia
GPs: General practitioners
HBV: Hepatitis B virus
HCC: Hepatocellular carcinoma
HL7: Health level 7
NASH: National Authentication Service for Health
PDF: Portable document format
PKI: Public key infrastructure
SMS: Short message service
SVHM: St Vincent’s Hospital Melbourne
